# The valproic acid rat model of autism presents with gut bacterial dysbiosis similar to that in human autism

**DOI:** 10.1186/s13229-018-0251-3

**Published:** 2018-12-10

**Authors:** Fang Liu, Kayla Horton-Sparks, Vanessa Hull, Robert W. Li, Verónica Martínez-Cerdeño

**Affiliations:** 1 College of Food Science and Engineering, Ocean University of China, Qingdao, China; 2 Institute for Pediatric Regenerative Medicine and Shriners Hospitals for Children Northern California, 2504 Stockton Blvd, Sacramento, CA 95817 USA; 3 United States Department of Agriculture, Agriculture Research Service (USDA-ARS), Animal Genomics and Improvement Laboratory, Beltsville, MD USA; 4 Department of Pathology and Laboratory Medicine, UC Davis School of Medicine, Sacramento, CA USA; 5 MIND Institute, Sacramento, CA USA

## Abstract

**Background:**

Gut microbiota has the capacity to impact the regular function of the brain, which can in turn affect the composition of microbiota. Autism spectrum disorder (ASD) patients suffer from gastrointestinal problems and experience changes in gut microbiota; however, it is not yet clear whether the change in the microbiota associated with ASD is a cause or a consequence of the disease.

**Methods:**

We have investigated the species richness and microbial composition in a valproic acid (VPA)-induced rat model autism. Fecal samples from the rectum were collected at necropsy, microbial total DNA was extracted, 16 rRNA genes sequenced using Illumina, and the global microbial co-occurrence network was constructed using a random matrix theory-based pipeline. Collected rat microbiome data were compared to available data derived from cases of autism.

**Results:**

We found that VPA administration during pregnancy reduced fecal microbial richness, changed the gut microbial composition, and altered the metabolite potential of the fecal microbial community in a pattern similar to that seen in patients with ASD. However, the global network property and network composition as well as microbial co-occurrence patterns were largely preserved in the offspring of rats exposed to prenatal administration of VPA.

**Conclusions:**

Our data on the microbiota of the VPA rat model of autism indicate that this model, in addition to behaviorally and anatomically mimicking the autistic brain as previously shown, also mimics the microbiome features of autism, making it one of the best-suited rodent models for the study of autism and ASD.

**Electronic supplementary material:**

The online version of this article (10.1186/s13229-018-0251-3) contains supplementary material, which is available to authorized users.

## Introduction

The gut and brain form the gut-brain axis through bidirectional nervous, endocrine, and immune communication. A change in one of these systems will most certainly have effects on the other systems. Disorders in the composition and quantity of gut microbiota can affect both the enteric nervous system and the central nervous system [1]. Specifically, microbiota has the capacity to impact the regular function of the brain, which can in turn affect the composition of microbiota via specific substances. Specific molecules and metabolic pathways in microbiota have been shown to be linked to neural development and neurodegenerative disorders, including Parkinson’s disease, Alzheimer’s disease, Huntington’s disease, schizophrenia, and multiple sclerosis [1–3].

Valproic acid (VPA) is a medication used for epilepsy and mood swings. Children prenatally exposed to VPA have an increased chance of being diagnosed with autism [4–7]. In addition, VPA exposure leads to accelerated or early brain growth which also occurs in some cases of autism [8]. Most importantly, VPA causes an alteration in the excitation/inhibition the cerebral cortex. Specifically, rats exposed to VPA in utero present with an increased glutamatergic and a decreased GABAergic component in the cortex [9]. The VPA rat model of autism experiences behavioral, immune, and microbiota changes similar to those described in patients with autism. We recently discovered that specific GABAergic interneuron types, the parvalbumin (PV)+ Chandelier (Ch) and PV+ Baskets cells (Bsk) cells, are decreased in the prefrontal cortex in autism [10, 11]. We also demonstrated that when VPA is administered via intraperitoneal injection to pregnant rats at a specific day of prenatal development with a specific dose (E (embryonic day) 12.5, 400 mg/kg), the offspring of these rats (“400-E12 VPA rats”) experienced a decrease in the number of PV+ Ch and PV+ Bsk cells in their adult cerebral cortex similar to what we found in humans with autism (under revision). In addition, the 400-E12 VPA rats also experienced behavioral changes similar to those exhibited by patients with autism (under revision).

ASD patients suffer from gastrointestinal problems and experience changes in the gut microbiota, including shifts in levels of *Firmicutes*, *Bacteroidetes*, *and Proteobacteria* with the abundance of *Lactobacillares* and *Clostridia* [12, 13]. Other gut commensals found to be altered in autism belong to the genera such as *Bifidobacterium*, *Lactobacillus*, *Prevotella*, and *Ruminococcus* [14]. Microbiome changes have been also described in several mouse models for autism, with one publication in a VPA mouse indicating a decreased abundance for *Bacteroidetes* in VPA exposed offspring [15]. It is not yet clear whether the changes in the microbiome associated to specific disease states are a cause or a consequence of the disease. Recent studies indicate that gut microbiota transplantation can transfer behavioral phenotypes, suggesting that the gut microbiota may be a modifiable factor modulating the development or pathogenesis of neuropsychiatric conditions. In this study, we investigated changes in microbial richness and microbiome composition in rats in response to VPA prenatal administration (400 mg/kg at E12) and found VPA-induced alterations similar to those seen in autism.

## Results

### VPA reduces fecal microbial richness of the offspring

A single IP injection of VPA during pregnancy in rats had a significant effect on fecal microbial richness in their offspring (*P* < 0.05, the Welch *t* test). In the control rats, Chao1 value was 1005.62 ± 120.00 (*N* = 11). VPA injection significantly reduced Chao1 to 925.98 ± 76.62 (*N* = 10, *P* < 0.05). However, other microbial diversity indicators, such as Pielou’s evenness, PD whole tree, and Shannon and Simpson indices, remained unchanged by VPA.

**Fig. 1:**
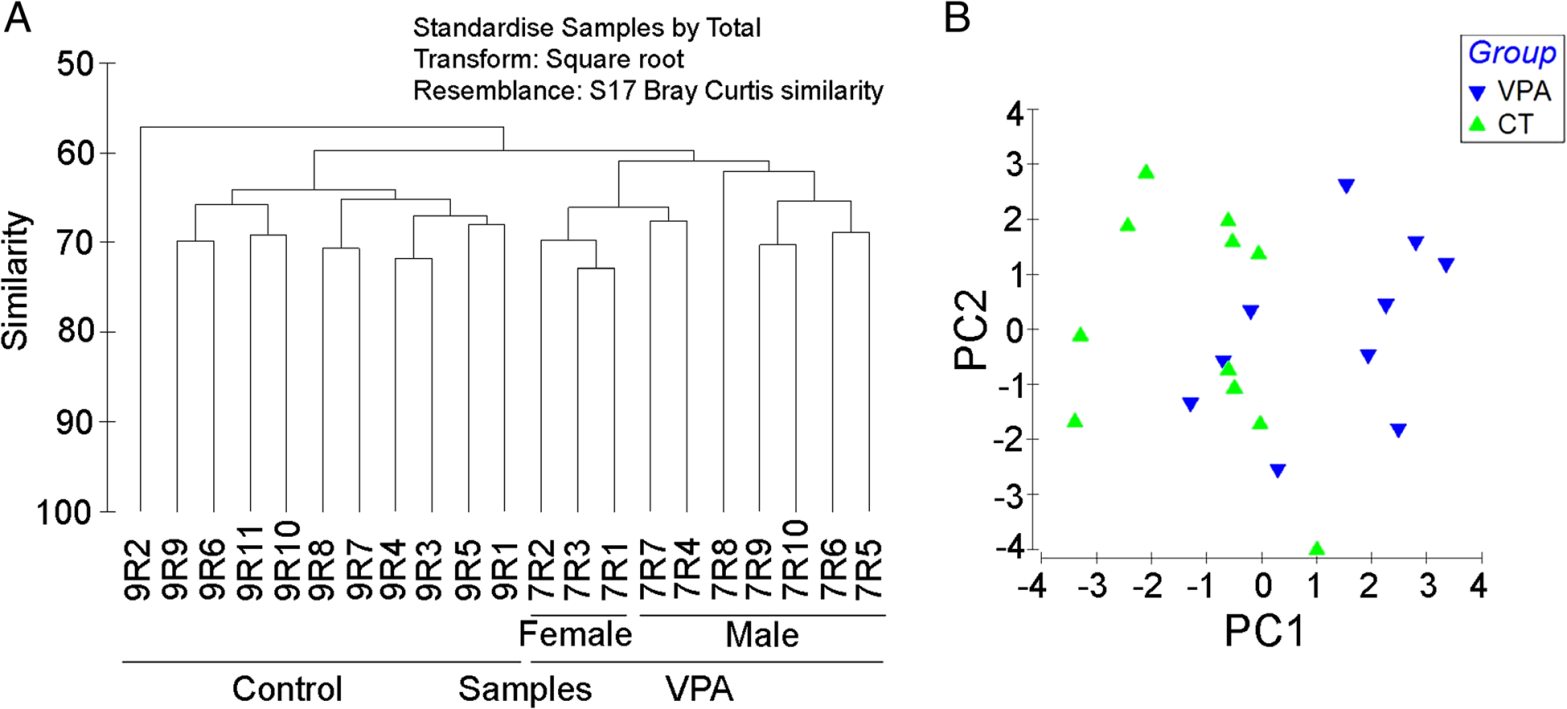
β-diversity in the gut microbial community of rats with or without prenatal valproic acid (VPA) exposure. **a** Clustering analysis based on Bray-Curtis similarity. Bray-Curtis similarity matrix based on square-root-transformed abundance at an OTU level. **b** Principal component analysis (PCA) based on Bray-Curtis similarity generated using the Vegan package in the R program. Control: rats without prenatal VPA exposure (*N* = 11). VPA: rats with VAP exposure (*N* = 10)

In utero VPA exposure also had a profound impact on fecal microbial structure. At the operational taxonomic unit (OTU) level, mean Bray-Curtis similarity values (%) within either the control or VPA groups were 63.57 ± 4.04, a significantly higher than mean similarity between the control and VPA groups (59.52 ± 3.24; *P* = 1.78 × 10^−12^). A cluster analysis using the group average approach of the resemblance values suggested individual microbial communities from the control and VPA groups were able to form two distinct clusters, respectively (Fig. 1). Together, our findings suggest that the effect of VPA may be long-lasting and could have a significant impact on the fecal microbial community structure in rats prenatally exposed to the toxin.

### VPA affects the gut microbial composition

**Fig. 2:**
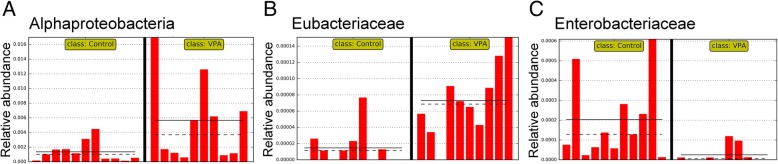
Microbial taxa displaying significant differences in relative abundance between rats with and without VPA exposure. **a** The Class Alpha-Proteobacteria. **b** The family Eubacteriaceae. **c** The family Enterobacteriaceae. Straight line, group mean abundance; dotted line, median. Control: rats without prenatal VPA exposure (*N* = 11). VPA, rats with VPA exposure (*N* = 10)

**Fig. 3:**
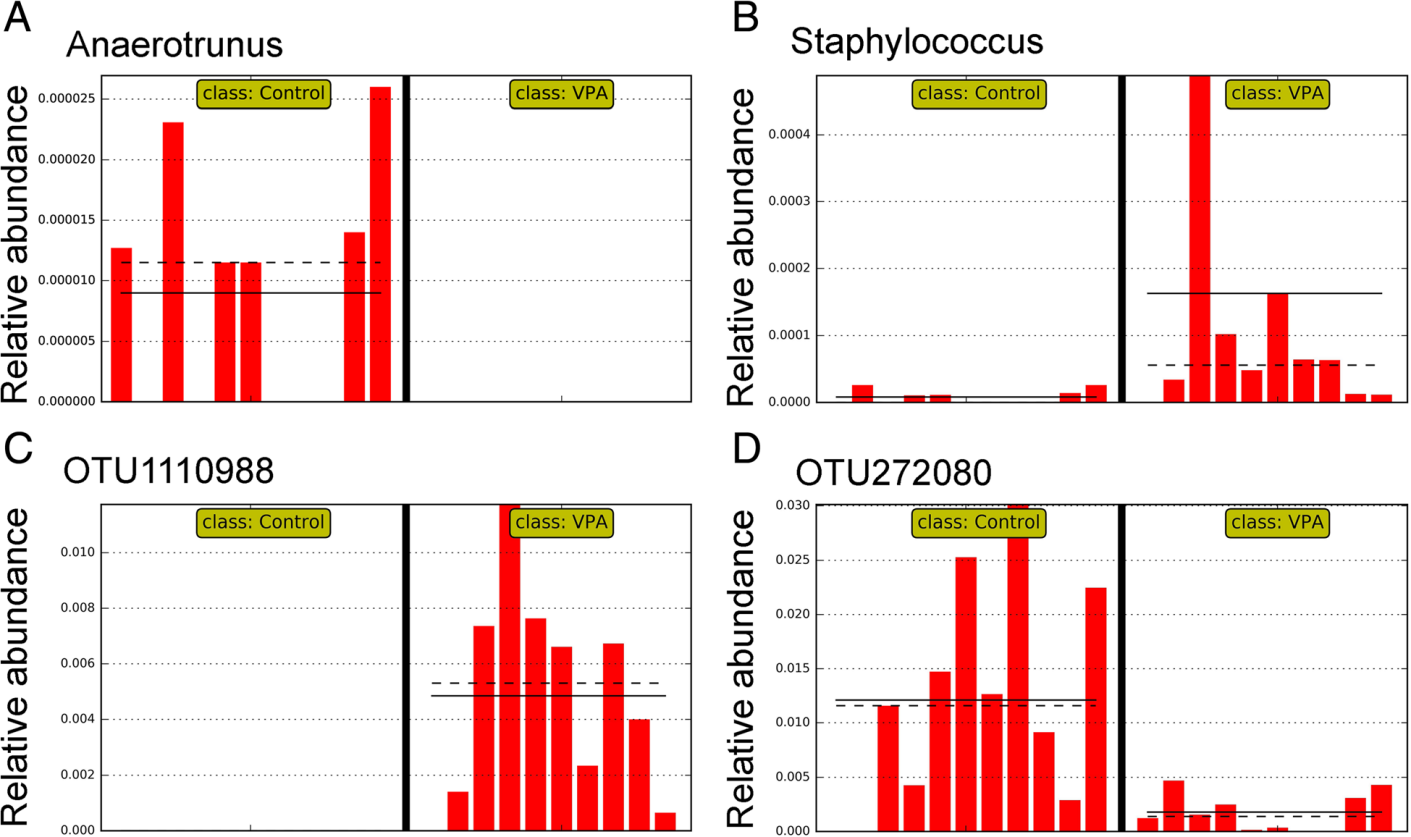
Select microbial genera and species with significant differences in relative abundance between rats with and without prenatal VPA exposure. **a** *Anaerotrunus*. **b** *Staphylococcus*. **c** OTU assigned to *Ruminococcus flavefaciens* (GreenGene ID# 1110988). **d** An OTU belonging to the family Lachnospiraceae (GreenGene ID# 272080). Straight line; group mean abundance; dotted line, median. Control: rats without prenatal VPA exposure (*N* = 11). VPA: rats with VPA exposure (*N* = 10)

Compared to the control group, VPA treatment significantly altered the abundance of 13 higher level taxa based on linear discriminating analysis (LDA) scores (the absolute log_10_ LDA score, or LDA, > 2.0 and *P* < 0.05 based on the Kruskal-Wallis test), including one class (α-Proteobacteria, Fig. 2a), four families (Fig. 2b, c), and six genera (Fig. 3a, b). For example, the abundance of α-Proteobacteria was significantly increased by VPA treatment (Fig. 2a; LDA > 3.4 and *P* < 0.05). The abundance of three families, Eubacteriaceae (Fig. 2b), Rikenellaceae, and Staphylococcaceae was also significantly increased by VPA (LDA > 2.0 and *P* < 0.05). On the other hand, the abundance of Enterobacteriaceae (Fig. 2c) was significantly repressed by VPA (LDA = 2.0229 and *P* = 0.0014). At the genus level, a significantly higher abundance level of the genus *Anaerotruncus* (Fig. 3a) was observed in the control group than in the VPA group while the VPA significantly increased the abundance of *Allobaculum*, *Anaerofustis*, *Proteus*, and *Staphylococcus* (LDA > 2.0 and *P* < 0.01; Fig. 3b).

The abundance of at least 100 OTU was significantly impacted by VPA treatment (LDA > 2.0 and *P* < 0.05 based on the Kruskal-Wallis test), representing approximately 10% of all OTU in a given gut microbial community (Additional file 1). Together, the relative abundance of these OTU accounted for approximately 15% of the fecal microbial community. Intriguingly, 93 of the 100 OTU significantly impacted by VPA belonged to the class Clostridia. Select OTU with significantly altered relative abundance by VPA were listed in Table 1. Compared to untreated controls, VPA repressed the abundance of 61 OTU while increasing that of 39 OTU. For example, 2 OTU assigned to a named species, *Ruminococcus flavefaciens*, ID_1110988 (Fig. 3c) and ID_562599, were significantly increased by VPA (Fig. 3c). Moreover, VPA had a profound impact on some of the most predominant OTU. Two OTU, ID_4296216 and ID_264734, belonging to the genus *Ruminococcus* and the family S24-7, respectively, were significantly increased by VPA; and both had relative abundance greater than 1.0%. OTU ID_272080 (Clostridiales, Fig. 3d) and ID_177930 (Lachnospiraceae) were also among the most abundant.

**Table 1:** Select OTUs significantly impacted by prenatal VPA injection

OTU_ID	Control	VPA	LDA	Annotation
513,445	0.31 ± 0.19	0.56 ± 0.29	3.0954	Bacteroidetes; Bacteroidia; Bacteroidales; Bacteroidaceae; Bacteroides
264,734	0.74 ± 0.54	1.51 ± 1.25	3.6429	Bacteroidetes; Bacteroidia; Bacteroidales; S24-7
581,474	0.00 ± 0.00	0.23 ± 0.33	3.0596	Firmicutes; Bacilli; Lactobacillales; Lactobacillaceae; Lactobacillus
272,080	1.21 ± 1.03	0.18 ± 0.18	3.7107	Firmicutes; Clostridia; Clostridiales
661,055	0.01 ± 0.01	0.54 ± 1.09	3.4725	Firmicutes; Clostridia; Clostridiales
1,110,312	0.17 ± 0.07	0.72 ± 0.51	3.4043	Firmicutes; Clostridia; Clostridiales
276,770	0.51 ± 0.40	0.03 ± 0.07	3.3477	Firmicutes; Clostridia; Clostridiales
631,564	0.44 ± 0.57	0.01 ± 0.01	3.2945	Firmicutes; Clostridia; Clostridiales
276,777	0.45 ± 0.46	0.12 ± 0.20	3.2476	Firmicutes; Clostridia; Clostridiales
460,611	0.48 ± 0.41	0.18 ± 0.31	3.1188	Firmicutes; Clostridia; Clostridiales
461,487	0.26 ± 0.16	0.52 ± 0.27	3.1123	Firmicutes; Clostridia; Clostridiales
290,338	0.35 ± 0.47	0.15 ± 0.25	3.0820	Firmicutes; Clostridia; Clostridiales
408,877	0.25 ± 0.35	0.03 ± 0.02	3.0675	Firmicutes; Clostridia; Clostridiales
348,404	0.10 ± 0.08	0.31 ± 0.19	3.0249	Firmicutes; Clostridia; Clostridiales
422,727	0.32 ± 0.19	0.12 ± 0.13	3.0146	Firmicutes; Clostridia; Clostridiales
277,208	0.02 ± 0.03	0.22 ± 0.22	3.0105	Firmicutes; Clostridia; Clostridiales
421,893	0.04 ± 0.14	0.27 ± 0.48	3.0056	Firmicutes; Clostridia; Clostridiales
310,760	0.12 ± 0.24	0.84 ± 0.78	3.5307	Firmicutes; Clostridia; Clostridiales; Lachnospiraceae
350,447	0.57 ± 0.46	0.17 ± 0.18	3.2725	Firmicutes; Clostridia; Clostridiales; Lachnospiraceae
177,930	0.83 ± 0.27	0.57 ± 0.30	3.1974	Firmicutes; Clostridia; Clostridiales; Lachnospiraceae
383,971	0.50 ± 0.34	0.21 ± 0.18	3.1159	Firmicutes; Clostridia; Clostridiales; Lachnospiraceae; [Ruminococcus]; gnavus
401,384	0.55 ± 0.29	0.27 ± 0.09	3.1489	Firmicutes; Clostridia; Clostridiales; Ruminococcaceae; Oscillospira
461,795	0.04 ± 0.04	0.27 ± 0.27	3.1198	Firmicutes; Clostridia; Clostridiales; Ruminococcaceae; Oscillospira
4,296,216	1.07 ± 0.71	2.40 ± 1.66	3.8102	Firmicutes; Clostridia; Clostridiales; Ruminococcaceae; Ruminococcus
268,043	0.54 ± 0.52	0.16 ± 0.16	3.2365	Firmicutes; Clostridia; Clostridiales; Ruminococcaceae; Ruminococcus
1,110,988	0.00 ± 0.00	0.48 ± 0.38	3.4032	Firmicutes; Clostridia; Clostridiales; Ruminococcaceae; Ruminococcus; flavefaciens

**Fig. 4:**
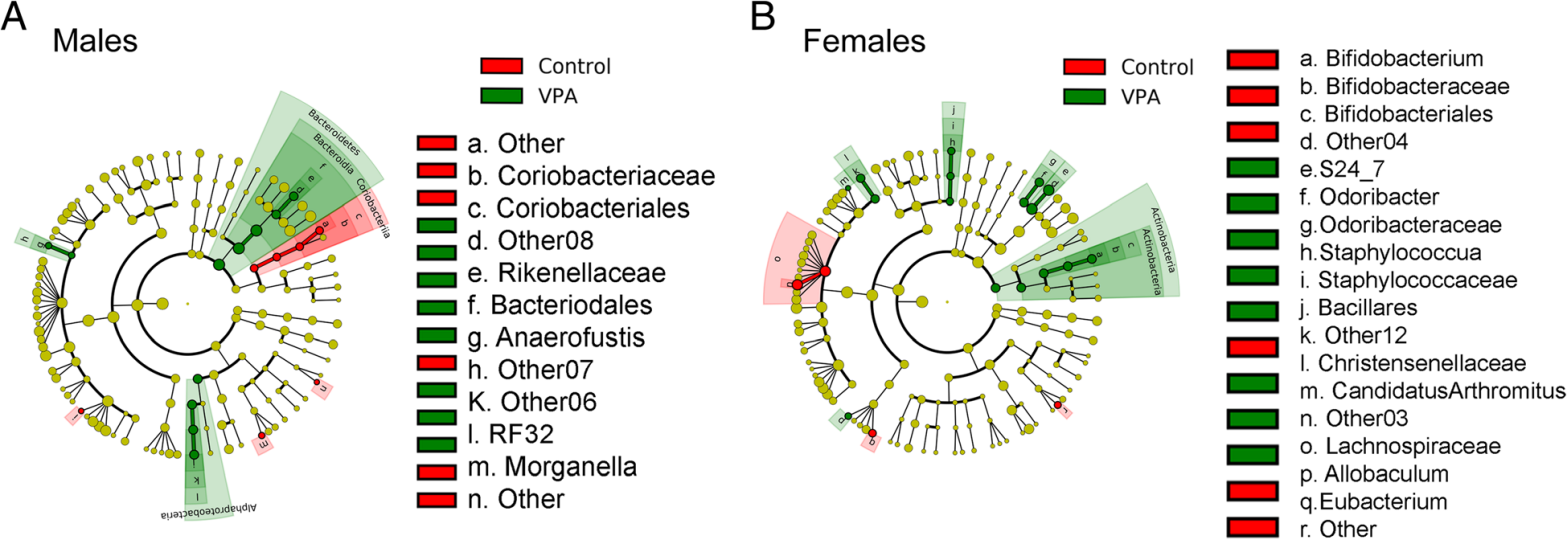
Graphical representation of the taxa with significantly different abundance in the gut microbial community of rats induced by prenatal VPA exposure. **a** Male rats with prenatal VPA exposure (VPA) comparing to male rats without prenatal VPA exposure (Control). **b** Female rats with prenatal VPA exposure (VPA) comparing to female rats without prenatal VPA exposure (Control). The statistical significance cutoff: absolute linear discriminant analysis (LDA) score log_10_ ≥ 2.0

Differences in microbial composition between the sexes were investigated by comparing male and female rats prenatally exposed to VPA with same-sex control rats. While uneven sample size in the male and female comparison may be a concern, the drastic sex-dependent changes induced by VPA were evident (Fig. 4a, b). At the phylum level, the abundance of Bacteroidetes was significantly increased by VPA in males only (LDA = 4.69; *P* < 0.05) while the abundance of Actinobacteria was significantly increased by VPA in females only (LDA = 3.50; *P* < 0.05), as compared to controls of the same sex. VPA significantly repressed the abundance of the class Coriobacteriia while it increased the two classes Bacteroidia and α-Proteobacteria in males only (LDA > 2.0 and *P* < 0.05). The abundance of several genera was significantly increased by VPA only in females, including *Allobaculum*, *Bifidobacterium*, *Odoribacter*, and *Staphylococcus* (LDA > 2.6 and *P* < 0.05). Intriguingly, the abundance of the genus *Candidatus* Arthromitus, a group of the segmented filamentous bacteria (SFB), was also significantly increased by VPA in female rats (LDA = 3.774 and *P* = 0.015) but not males. There is strong evidence demonstrating that these gut epithelium-associated bacteria possess strong abilities to modulate host immune responses.

At the species (OTU) level, VPA prenatal exposure induced significant changes in the relative abundance of 66 and 72 OTU in male and female rats, respectively. Among them, the abundance of 61 OTU was also significantly impacted by VPA exposure regardless of gender. A total of 9 OTU displayed significant directional changes by VPA in both male and female rats (Table 2). For example, the relative abundance of an OTU (GreenGene ID_1110312) assigned to the order Clostridiales and an OTU (GreenGene ID_1110988) assigned to *Ruminococcus flavefaciens* was significantly higher in both male and female rats with prenatal VPA exposure (LDA > 3.40; *P* < 0.001) while 7 other OTU were significantly reduced in fecal microbial communities of both male and female rats with VPA exposure (LDA > 2.0 and *P* < 0.05).

**Table 2:** Nine OTUs displayed a significant difference in relative abundance between control and VPA groups regardless of sex. The numbers denote relative abundance (mean ± SD)

OTU_ID	Control	VPA	LDA value	Annotation
50,208	0.0316 ± 0.0313	0.0000 ± 0.0000	2.1609	Clostridiales
227,788	0.0845 ± 0.0743	0.0000 ± 0.0000	2.6365	Clostridiales
321,960	0.0477 ± 0.0235	0.0000 ± 0.0000	2.3910	Clostridiales; Ruminococcaceae
352,799	0.1134 ± 0.1965	0.0091 ± 0.0059	2.7102	Clostridiales; Lachnospiraceae
450,576	0.0502 ± 0.0400	0.0000 ± 0.0000	2.4470	Clostridiales; Ruminococcaceae
839,137	0.0493 ± 0.0325	0.0215 ± 0.0146	2.1805	Clostridiales; Lachnospiraceae
1,107,799	0.0524 ± 0.0431	0.0073 ± 0.0102	2.3709	Clostridiales
1,110,312	0.1708 ± 0.0684	0.7212 ± 0.5143	3.4043	Clostridiales
1,110,988	0.0001 ± 0.0004	0.4848 ± 0.3770	3.4032	Clostridiales; Ruminococcaceae; Ruminococcus; flavefaciens

### VPA alters the metabolite potential of the fecal microbial community

Among the 5264 predicted KEGG proteins from the rat fecal microbiome, 4331 proteins were supported by at least 10 hits. Several proteins belonging to ABC transporters, such as multiple sugar transport system permease protein (K02025) and ATP-binding cassette, subfamily B, bacterial (K06147), and RNA polymerase sigma-70 factor, ECF subfamily (K03088) were among the most abundant. Compared to the control, VPA injection repressed the abundance of 11 KEGG proteins, including putative ABC transport system ATP-binding protein (K02003), multiple sugar transport system substrate-binding protein (K02027), LacI family transcriptional regulator (K02529), methyl-accepting chemotaxis protein (K03406), two proteins related to two-component system, K07718 and K07720, and four proteins in the peptide/nickel transport system (K02031, K02032, K02033, K02034; ATP-binding and permease proteins, respectively).

VPA injection appeared to have a profound impact on gut microbial metabolic pathways. A total of 29 pathways were significantly impacted by VPA (LDA score > 2.0; *P* < 0.05), resulting in a significantly elevated hit count for 21 pathways while repressing 8 pathways (Table 3). For example, the normalized hit counts assigned to bacterial secretion system, DNA replication, DNA repairs and recombination proteins, histidine metabolism, and lipid biosynthesis were significantly increased by VPA. On the other hand, ABC transporters, the most abundant pathways in numerous biological systems, and two-component system, bacterial chemotaxis and bacterial motility proteins, were significantly repressed by VPA.

**Table 3:** The microbial pathways significantly impacted by VPA

Pathways	Control	VPA	LDA score	*P* value
ABC transporters	3.29 ± 0.39	3.04 ± 0.24	3.0834	0.0486
Amino acid-related enzymes	1.41 ± 0.01	1.43 ± 0.02	2.0357	0.0039
Bacterial chemotaxis	0.57 ± 0.12	0.47 ± 0.07	2.6664	0.0346
Bacterial motility proteins	1.23 ± 0.27	1.01 ± 0.15	3.0238	0.0411
Bacterial secretion system	0.48 ± 0.02	0.50 ± 0.03	2.1010	0.0167
Carbon fixation pathways in prokaryotes	0.95 ± 0.09	1.03 ± 0.07	2.5883	0.0201
Chaperones and folding catalysts	0.96 ± 0.06	1.01 ± 0.04	2.3375	0.0411
Chromosome	1.51 ± 0.03	1.53 ± 0.02	2.1418	0.0137
Citrate cycle	0.54 ± 0.08	0.62 ± 0.07	2.5479	0.0167
DNA repair and recombination proteins	2.68 ± 0.06	2.75 ± 0.05	2.5379	0.0167
DNA replication	0.62 ± 0.02	0.64 ± 0.02	2.0466	0.0112
DNA replication proteins	1.17 ± 0.03	1.20 ± 0.03	2.2293	0.0346
Flagellar assembly	0.61 ± 0.15	0.48 ± 0.09	2.7850	0.0486
Glycine, serine and threonine metabolism	0.80 ± 0.02	0.82 ± 0.02	2.0011	0.0290
Histidine metabolism	0.63 ± 0.04	0.66 ± 0.02	2.1867	0.0242
Homologous recombination	0.89 ± 0.03	0.93 ± 0.02	2.2693	0.0112
Lipid biosynthesis proteins	0.59 ± 0.03	0.61 ± 0.02	2.0892	0.0486
Oxidative phosphorylation	1.01 ± 0.08	1.08 ± 0.08	2.5044	0.0411
Peptidases	1.90 ± 0.04	1.93 ± 0.03	2.1605	0.0486
Porphyrin and chlorophyll metabolism	0.95 ± 0.04	0.89 ± 0.05	2.4393	0.0201
Protein export	0.55 ± 0.02	0.57 ± 0.02	2.1048	0.0167
Protein kinases	0.34 ± 0.03	0.31 ± 0.02	2.1524	0.0242
Pyrimidine metabolism	1.79 ± 0.03	1.82 ± 0.02	2.2223	0.0242
Riboflavin metabolism	0.19 ± 0.03	0.21 ± 0.02	2.0135	0.0486
Ribosome	2.13 ± 0.07	2.21 ± 0.07	2.6423	0.0167
Terpenoid backbone biosynthesis	0.51 ± 0.02	0.53 ± 0.02	2.0739	0.0201
Transporters	7.52 ± 1.02	6.79 ± 0.66	3.5543	0.0290
Two-component system	1.59 ± 0.13	1.49 ± 0.06	2.6900	0.0290

### Microbial co-occurrence patterns and network structure remain unchanged by VPA

**Fig. 5:**
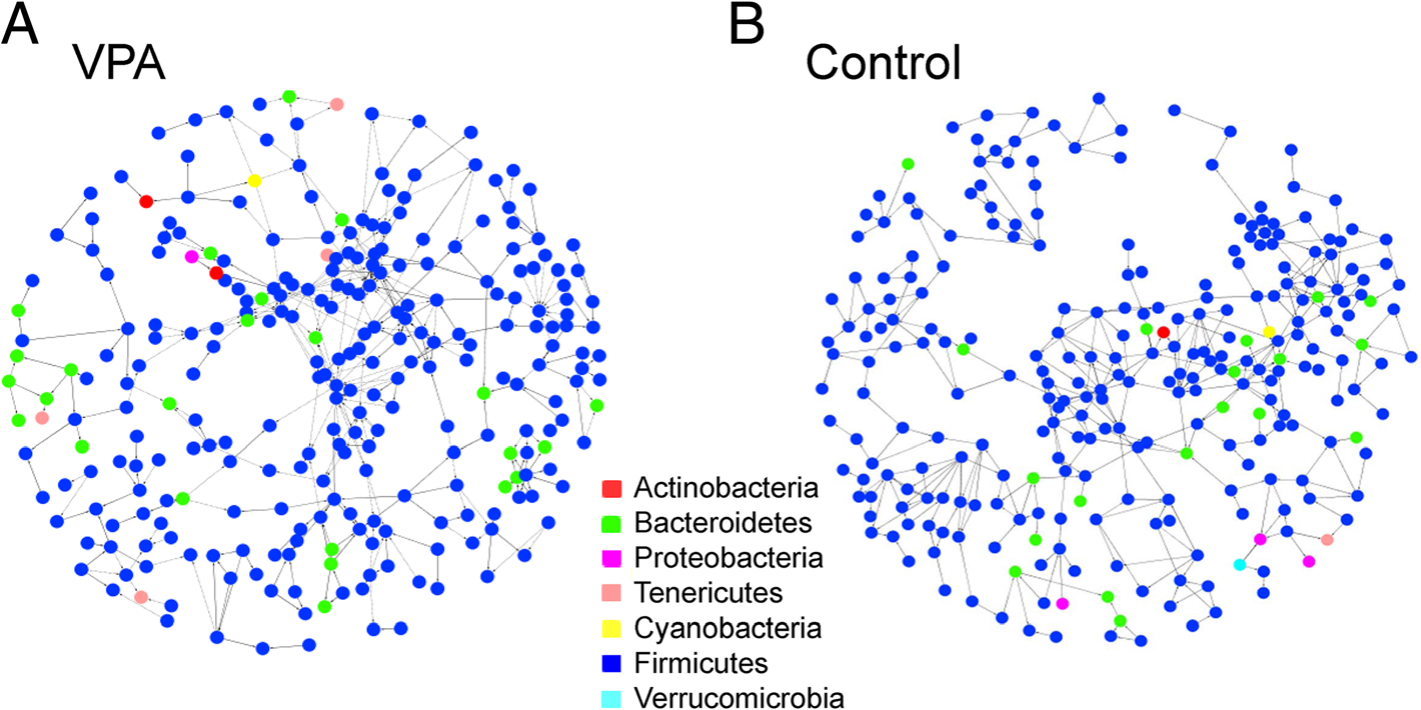
Visualization of microbial co-occurrence networks identified using the fast greedy modularity optimization method in the rats with and without prenatal VPA exposure. **a** The rats with prenatal VPA exposure (VPA). **b** Control rats without prenatal VPA exposure. Nodes represent an OTU. Edge (links) with solid lines, positive connection; dashed lines, negative connection. The color of the nodes indicates the phylum to which the OTU belong

As Table 4 shows, the global network properties as well as network composition and microbial co-occurrence patterns in fecal microbial communities of the offspring between the control and VPA-treated rats were largely indistinguishable. Both global networks were highly modular with a modularity between 0.84 and 0.86. Both networks shared 230 nodes (OTU) or 57.1% of all members. The number of large modules with ≥ 10 members in the two networks was identical [12]. Moreover, the relative proportion (%) of OTU node distributions at the phylum level was stable between the two networks (Fig. 5). For example, the most dominant phylum in both networks were Firmicutes, accounting for 89.6% and 87.6% of all OTU in the control and VPA networks, respectively, which was similar to the percentage of the OTU assigned to Firmicutes in the microbial communities prior to network inference (88.3 and 87.5%, in the control and VPA groups, respectively). Moreover, the percentage of OTU nodes assigned to Actinobacteria was 0.50 and 0.49% in the control and VPA networks, respectively. Some minor yet notable differences existed, nevertheless. The percentage of OTU nodes assigned to Proteobacteria was 0.99% and 0.49% in the control and VPA networks, respectively. Of note, one OTU (GreenGeneID_1136443) assigned to *Mucispirillum schaedleri*, the sole species in the phylum Deferribacteres, was present in every sample collected in a relatively high abundance but did not interact with any other OTU in the communities. As a result, this species was not a member of either network.

**Table 4:** Select topological properties of global networks of fecal microbial communities of the offspring of rats with prenatal administration of PBS (Control) and VPA

Network property	Control	VPA
Total nodes	403	411
Total links	487	488
Modularity	0.856	0.843
Number of modules	55	60
Number of modules with ≥ 10 members	12	12
Average degree (avgK)	2.417	2.375
Average clustering coefficient (avgCC)	0.138	0.087
Average path distance (GD)	9.579	7.169
Geodesic efficiency (*E*)	0.148	0.185
Harmonic geodesic distance (HD)	6.772	5.398
Maximal degree	13	16
Centralization of degree (CD)	0.026	0.033
Maximal eigenvector centrality	0.268	0.374
Centralization of eigenvector centrality (CE)	0.249	0.359
Density (*D*)	0.006	0.006
Transitivity (Trans)	0.187	0.117
Connectedness (Con)	0.464	0.340

**Fig. 6:**
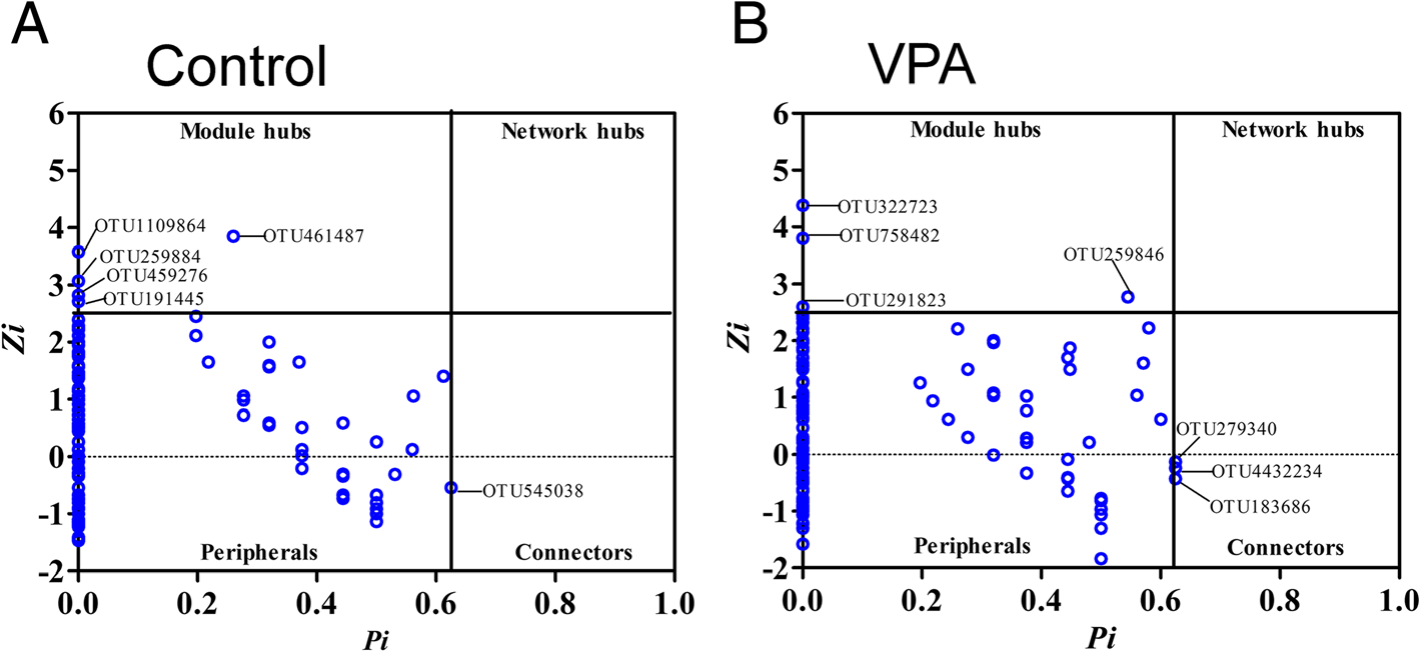
The scatter plot showing the distribution of OTU based on their topological roles in the network in the gut microbial community of rats with and without prenatal VPA exposure. **a** Control. **b** VPA. Each dot represents an OTU. Z, within-module connectivity. P, Among-module connectivity

The Z-P scatter plots allowed us to dissect the topological roles of OTU nodes in the network and infer their possible ecological function in the fecal microbial community. As Fig. 6 shows, > 98% of the OTU nodes in both networks were peripherals with most of their links lying inside their own modules, based on the Olesen classification [16]. These OTU likely acted as specialists in the microbial community. A total of six OTU, all assigned to the order Clostridiales, may function as generalists in the fecal microbial community of control rats, including one OTU (GreenGene ID_545038), assigned to the family Peptostreptococcaceae, acted as a connector species, linking modules together while other five OTU were module hubs and may play an important role for the coherence of its own module. The relative abundance of the two of the five OTU, GreenGene_ID_461487 and _1109864, was also significantly altered by VPA administration. In the VPA network, the OTU acted as connectors and module hubs were completely different. While all three connectors were from the order Clostridiales, two of them belonged to the family Ruminococcaceae (GreenGene ID_183686 and _4432234). On the other hand, one of the four module hubs, GreenGene ID_322723, was from the genus *Lactobacillus* while other three OTU were from the order Clostridiales in the VPA network. Overall, we demonstrated that prenatal administration of VPA reduces fecal microbial richness, changes the gut microbial composition, and alters the metabolite potential of the fecal microbial community in rats. However, the global network property and network composition as well as microbial co-occurrence patterns are largely preserved in these animals.

## Materials and methods

### VPA administration

Intraperitoneal administration of VPA (valproic acid sodium salt, Sigma P4543) was delivered to pregnant Sprague Dawley rats (8 weeks old) at E12.5 (*n* = 3). Pregnant control dams of the same age were injected with sterile saline also at E12.5 (*n* = 5). The pups of these dams were the subjects of this study. We collected stool and tissue samples from 10 VPA offspring and 11 control offspring equally distributed among groups.

### Fecal total DNA extraction

Fecal samples from the rectum were collected from 8-week-old rats at necropsy and snap-frozen in liquid nitrogen and stored at − 80 °C freezers until total DNA was extracted. Microbial total DNA was extracted from fecal samples using a QIAamp PowerFecal DNA Kit (Qiagen, Germantown, MD, USA). DNA integrity and concentration were quantified using a BioAnalyzer 2100 (Agilent, Palo Alto, CA, USA).

### Illumina sequencing of 16S rRNA genes

The 16S rRNA gene sequencing was performed as previously described [17, 18]. The hypervariable V3–V4 regions of the 16S rRNA gene were directly amplified from 20 ng of input total DNA using Illumina platform-compatible PAGE-purified adaptor oligos that contain important features including sequencing primers, sample-specific barcodes, and 16S PCR primers (forward primer, 341/357F, CCTACGGGNGGCWGCAG; reverse primer, 805R: GACTACHVGGGTATCTAATCC). The PCR reaction included 1.25 units of AccuPrime TaqDNA Polymerase High Fidelity (Invitrogen, Carlsbad, CA, USA) in a 25-μl reaction buffer containing 200 nM primers, 200 nM dNTP, 60 mM Tris-SO4, 18 mM (NH4)_2_SO4, 2.0 mM MgSO4, 1% glycerol, and 100 ng/uL bovine serum albumin (New England BioLabs, Ipswich, MA, USA). PCR was performed using the following cycling profile: initial denaturing at 95 °C for 2 min followed by 20 cycles of 95 °C 30 s, 60 °C 30 s, and 72 °C 60 s. Amplicons were purified using Agencourt AMPure XP bead kits (Beckman Coulter Genomics, Danvers, MA, USA) and quantified using a BioAnalyzer DNA 7500 chip kit and a QuantiFluor fluorometer. The purified amplicons from individual samples were pooled in equal molar ratios. The purified amplicon pool was further spiked with approximately 25% of whole-genome shotgun libraries prepared using an Illumina TruSeq DNA sample prep kit with a compatible adaptor barcode to enhance sequence diversity during the first few cycles of sequencing for better cluster differentiation. The concentration of the pooled final library pool was quantified using a BioAnalyzer high-sensitivity DNA chip kit (Agilent). The library pool was sequenced using an Illumina MiSeq Reagent Kit v3 on an Illumina MiSeq sequencer as described previously. The mean number of 2 × 250 bp pair-end sequences obtained was 347,849.14 (± 90,627.63, SD, *N* = 21) per sample.

### Sequence data analysis

The sequence data were preprocessed using MiSeq Control Software (MCS) v2.4.1. Raw sequences were first analyzed using FastQC version 0.11.2 to check basic statistics, such as GC%, per base quality score distribution, and sequences flagged as poor quality. The four maximally degenerate bases (NNNN) at the most 5′ end of the read pair, which were designed to maximize the diversity during the first four bases of the sequencing run for better identification of unique clusters and improve base-calling accuracy, were then removed. The presence of forward and reverse PCR primers at the 5′ and 3′ ends of each sequence read was scanned; the reads without primers were discarded. Chimeric reads were also removed. The processed pair-end reads were then merged using PandaSeq v2.8 to generate representative complete nucleotide sequences (contigs) using default parameters. The overlapping regions of the pair-end read were first aligned and scored, and reads with low score alignments and high rate of mismatches were discarded. After these quality control steps and filtering procedures, greater than 91% of the input raw sequences (mean 347,849 reads per sample) retained for subsequent analysis.

The QIIME pipeline (v.1.9.1) with the default reference v. 0.1.3 was used to analyze the 16S rRNA gene sequences. Both “closed reference” and “open reference” protocols in the pipeline were used for OTU picking as previously described [18]. The rarefaction depth was set to 100,000 quality reads per sample. The default QIIME parameters were used, except for that the OTU abundance threshold (lowered to 0.0001%). The GreenGene database (v13.8) was used for taxonomy assignment (greengenes.lbl.gov). PyNAST (v1.2.2) was used for sequence alignment. PICRUSt (v1.0.0), a software package designed to predict metagenome functional contents from marker gene surveys (Langille et al., 2013), was used with default parameters to predict gene contents and metagenomic functional information based on the OTU table generated using the closed-reference protocol in QIIME. Briefly, the OTU table was first normalized by dividing each OTU by the known/predicted 16S copy number by using the PICRUSt workflow: normalize_by_copy_number.py. The gene contents or the abundance of KEGG Orthology (KO) were predicted from the normalized OTU table using the workflow: predict_metagenomes.py. The predicted metagenome function was further analyzed by collapsing thousands of KEGG Orthologs into higher functional categories (pathways) (categorize_by_function.py). In addition, specific OTU contributing to a given function or pathway was identified by using the workflow: metagenome_contributions.py, as described previously [17]. The linear discriminant analysis effect size (LEfSe) algorithm was used to identify OTU relative abundance values and KEGG gene families and pathways that display significant differences between two biological conditions [19] with a default cutoff (the absolute log_10_ LDA score or LDA > 2.0 and *P* values < 0.05 based on the Kruskal-Wallis test by ranks).

### Network construction and visualization

The global microbial co-occurrence network was constructed using a random matrix theory (RMT)-based pipeline [20, 21]. The OTU detected in < 50% of all samples were excluded due to a drastic effect of OTU sparsity on the precision and sensitivity of network inference [22]. A similarity matrix, which measures the degree of concordance between the abundance profiles of individual OTU across different samples, was then obtained by using Pearson correlation analysis of the abundance data [20]. A threshold cutoff value (0.88) was automatically determined by calculating the transition from Gaussian orthogonal ensemble to Poisson distribution of the nearest-neighbor spacing distribution of eigenvalues, in the pipeline and then applied to generate an adjacent matrix for network inference [21]. The fast-greedy modularity optimization procedure was used for module separation. The within-module degree (Z) and among-module connectivity (P) were then calculated and plotted to generate a scatter plot for each network to gain insights into the topological roles of individual nodes in the network according to the Olesen classification [21]. The network structure was finally visualized using Cytoscape v3.6.1.

## Discussion

The gut and brain form the gut-brain axis through bidirectional nervous, endocrine, and immune communications. Mammalian species often contain similar microbiome richness at the level of phylum, but diversity and richness of species are highly variable among individuals [23]. This variability is determined by many factors, including genetics, environment, diet, disease, stress, and age [24]. When microbiota composition is altered due to any of these factors, the function of the intestinal mucosal barrier is reduced; and bacterial products such as amyloids and lipopolysaccharides leak, increasing the permeability of the blood brain barrier, which, in turn, affects the central nervous system [25].

Humans with autism and mice models of autism have shown significant alterations in their microbiota composition. Children with autism present with more GI symptoms than typically developing children, and the severity of their GI symptoms is correlated to the severity of their behavioral symptoms [26, 27]. These children also demonstrate bacterial dysbiosis, which has been suggested to play a role in autism’s etiology [28]. While different studies have found changes in specific bacteria are often associated to dysbiosis in autism, it is generally accepted that the gut microbial community of patients with autism displays a higher relative abundance of Lactobacillacease and Clostridia and a reduced incidence of the *Prevotella* and other fermenters [29–35].

Studies in mice have allowed to better understand the role of the microbiota in autism [36]. The lack of microbiota produces changes in behavior. For example, germ-free mice lack a preference for spending time with another mouse over spending time in an empty chamber and deviate from the experimental expectation that they would spend more time exploring a space containing a new mouse rather than a familiar mouse [37, 38]. Germ-free mice also show a differential gene expression associated with neuronal structure and function in the amygdala [39]. Germ-free rats present with a social deficit phenotype in the reciprocal social interaction test [40]. Antibiotic treatment in wildtype and mouse models of autism also affects social behavior [15, 41, 42]. On the other hand, the use of probiotics ameliorates behavioral deficits [38, 42]. Together, these data point out a role of microbiota in regulating behavior. The nature of microbiota has been studied in several mouse models for autism. The inbred mouse, BTBR, that presents with the full spectrum of ASD-like behavior, shows an overall decrease in bacterial diversity characterized by an increase in the relative abundance of the genus *Akkermansia* and a decrease in abundance of *Bifidobacterium and Clostridiales* [43–45]*.* In addition, BTBR mice have impaired intestinal integrity and a deficit in the intestinal tight junction proteins *Ocln* and *Tjp1* [46]. Environmental mice models of autism have also produced information about the importance of microbiota in this condition. In the maternal immune activation (MIA) mouse model, the species richness did not differ significantly between control and MIA offspring, but the offspring displayed decreased intestinal barrier integrity, altered gut microbiota, and increased abundance of the families Lachnospiraceae, Porphyromonadaceae, and Prevotellaceae [47]. In the maternal high-fat diet (MHFD) mouse model for autism, the diversity of the microbiota was decreased compared to the control group, with marked decreased in *Lactobacillus*, *Parabacteroides*, *Helicobacter*, and *B. uniformis*. In this study, we demonstrated that species richness in the fecal microbial community in the autistic-like rat model, the 400-E12 VPA rat, was significantly reduced. Using next-generation sequencing technology in a murine autism model, it was reported that the microbiome composition in mice in utero exposed to VPA presented with a decreased of *Bacteroid*s [15]. Other gut commensals found to be altered in the VPA mice were *Deltaproteobacteris* and *Erysipelotrichales*. These changes in VPA mouse microbiota composition were coincident with changes in behaviors linked to autism [15].

Our 400-E12 VPA rats showed a decrease in microbial diversity (species richness). Specifically, significant increases in the abundance of *α-Proteobacteria*, *Eubateriaceae*, *Rikenellaceae*, and *Staphylococcaceae*. On the other hand, Enterobacteriaceae was significantly decreased by VPA exposure in utero. At the genus level, we found a significantly higher abundance of the genus *Anaerotruncus* in the control group and a significantly increased abundance of the genera *Allobaculum*, *Anaerofustis*, *Proteus*, and *Staphylococcus* in the VPA group.

This is the first time the microbial species richness and microbiome composition have been studied in a rat model for autism, the 400-E12 VPA rat. The decrease in microbial diversity in this rat model was consistent with the observations in human autism and most of the mouse models of autism studied to date. The gut microbial composition was largely similar to that of humans with autism and murine autism-like models. The enteric bacteria, especially the class Clostridia, are known to play an important role in children with autism (Frye et al. 2015). In our study, Clostridia is the most dominant class in the rat fecal microbial community, accounting for more than 60% of all sequence reads, followed by the class Bacteroidia with more than 30% of the sequences. Among the 100 OTU significantly impacted by prenatal VPA administration, the vast majority of them, 94, belonged to Clostridia, suggesting that ecological manipulation via antibiotics or pre- or pro-biotic approaches targeting this class of gut bacteria may prove effective in alleviating autism symptoms. A significant reduction in microbial species richness, such as Chao1, in the 400-E12 VPA rats was consistent with the observation in BTBR T^+^Itpr3^tf^/J mouse model of autism [44]. However, biodiversity encompasses both species richness and evenness as well as interactions among species in the ecosystem [16]. While a marked reduction in species richness was evident in the rats with prenatal VPA exposure, species evenness in the rat gut microbial community did not appear to be impacted. Furthermore, the microbial co-occurrence patterns and microbial interactions in the community appeared to be preserved in the rats with prenatal VPA exposure.

Moreover, our findings provide further evidence of sex-specific alterations of gut microbiome by prenatal VPA administration in rodents [15]. For example, in male rats, the abundance of the family Coriobacteriaceae as well as the class Coriobacteriia was significantly repressed by VPA. An OTU (GreenGene ID_1113282), belonging to Mollicutes, was significantly increased by VPA. On the other hand, a twofold increase in the relative abundance of the phylum Proteobacteria, from 1.03% in the control rats to 2.17% in the male rats with VPA exposure, was observed. The VPA-induced increase became more evident in the class α-Proteobacteria, from 0.14% in the control male rats to 0.56% in the male rats with prenatal VPA exposure. The Proteobacteria are known to be a marker for an unstable microbial community and a risk factor of human disease [48, 49]. An elevated Proteobacteria level is frequently associated with metabolic disorders and intestinal inflammation. The pathological relevance of elevated Proteobacteria abundance in autism warrants further investigation. In contrast to male rats, prenatal VPA exposure induced a distinguishingly different set of microbial taxa in female rats. The abundance of the genus Staphylococcus and the family S24-7 was significantly increased by prenatal VPA exposure only in female rats. A significant elevation of Candidatus Arthromitus, which harbors commensal SFB, by VPA was observed only in female rats. Numerous studies have established solid links between SFB colonization and human disease [50]. As a potent inducer of IgA production and T_H_17 immune responses as well as innate immunity, SFB may play a role in the pathogenesis of autism. Indeed, a recent study shows that pregnant mice colonized with SFB were more likely to produce offspring with maternal immune activation (MIA)-associated abnormalities [41].

The composition of the microbiota is of great importance to the function of the brain. Bacteria can regulate brain function through several mechanisms. Some bacteria, such as *Bifidobacterium* and *Lactobacillus*, that inhabit in the gut, have the capacity to produce anti-inflammatory cytokines, while other, such as *Clostridium* and *Ruminococcus* [51], can produce pro-inflammatory cytokines*.* Metabolic products of the gut microbiota, such as short-chain fatty acids, have also been implicated in autism. Gut microbiota has been suggested to regulate many nervous functions including neurogenesis, differentiation, myelination, formation and integrity of the blood-brain barrier, neurotrophin and neurotransmitter release, apoptosis, gap junction modification, and synaptic pruning [52]. Moreover, several microRNAs participate in signaling networks through the intervention of the gut microbiota [53]. In addition, gut microbiota release inflammatory cytokines that can act as epigenetic regulators and regulate gene expression being a factor for example in cancer risk and diabetes-associated autoantigens [54–56]. Here, we demonstrated that VPA also alters the metabolite potential of the microbial community in rats. VPA prenatal administration significantly elevated 21 bacterial pathways while repressing 8 pathways. Among them, there was an increase in activation of the bacterial secretion system, DNA replication, DNA repairs, and recombination proteins and a decrease in ABC bacterial transporter pathways. These data indicate a potentially higher activity of those pathways related to bacterial survival and function.

In conclusion, our data on the gut microbial community of the 400-E12 rats in response to prenatal VPA exposure indicate that this model, in addition to demonstrating behavioral and anatomical similarities to autism, also mimics the microbiota features of autism, making it one of the best-suited rodent models for the study of autism.

## Additional file

Additional file 1: All OTU in a given gut microbial community. (XLSX 180 kb)

## Abbreviations

ASD: Autism spectrum disorder
Bsk: Baskets cells
Ch: Chandelier
LDA: Linear discriminating analysis
MHFD: Maternal high-fat diet
MIA: Maternal immune activation
OTU: Operational taxonomic unit
PV: Parvalbumin
RMT: Random matrix theory
SFB: Segmented filamentous bacteria
VPA: Valproic acid
